# Maternal care predicts facial expression processing in macaques

**DOI:** 10.1016/j.isci.2025.112179

**Published:** 2025-03-25

**Authors:** Olivia O’Callaghan, Jamie Whitehouse, Annika Paukner, Claire L. Witham, Bridget M. Waller

**Affiliations:** 1 Department of Psychology, Nottingham Trent University, Nottingham, UK; 2 Centre for Macaques, Medical Research Council, UK

**Keywords:** Biological sciences, Neuroscience, Behavioral neuroscience

## Abstract

Facial expressions are common across mammals and are essential for social communication. In humans, a rich early social environment is important for the appropriate development of facial expression processing. Whether other animals are similarly reliant on social input for facial expression development, or have a more fixed system, is unknown. Here, we investigated how maternal care influences facial expression processing skills in rhesus macaques (*Macaca mulatta*). We conducted three experiments quantifying facial expression processing and examined performance in relation to historical maternal data and across age. Facial expression processing skill was predicted by positive social contact with the mother during infancy and increased with age until adulthood. Our findings provide the first evidence that early social input, specifically maternal care, enhances facial expression processing skills in non-human animals. This challenges the notion that facial expression processing systems are entirely hard-wired and innate and emphasizes the importance of flexibility and responsiveness to local conditions.

## Highlights

• Macaques exhibit individual differences in facial expression processing
• Facial expression processing improves during infancy and stabilizes in adulthood
• Increased maternal care is associated with better facial expression processing
• Facial expression processing is subject to environmental influence

## Graphical abstract

**Figure:**
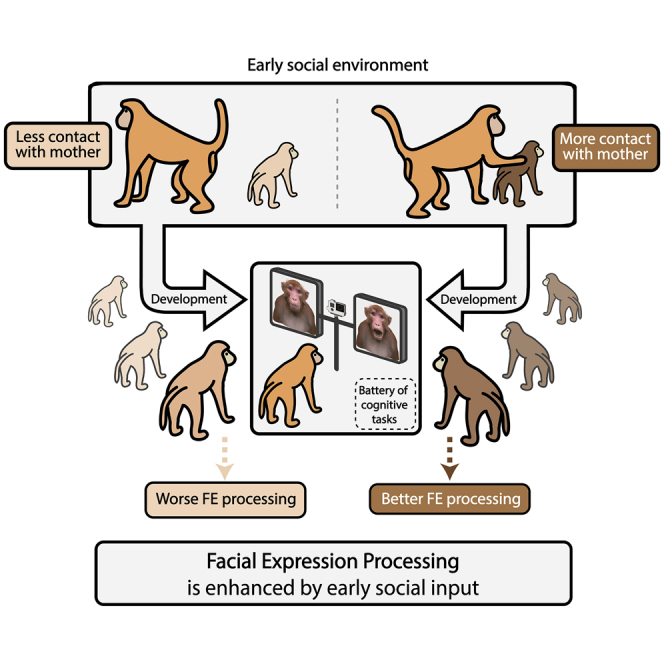

## Introduction

Facial expressions can convey information about an individual’s potential future actions, emotion, and social motives.^1^^,^^2^^,^^3^ The extent to which the production and perception of facial expressions are flexible and subject to environmental and cultural influences has been a long-standing debate in emotion theory.^4^^,^^5^ Many argue that facial expressions are largely innate and universal,^6^^,^^7^^,^^8^ with blind children producing similar expressions to sighted children^9^ and expressions being recognized cross-culturally^10^^,^^11^^,^^12^ supporting this view. This premise of hardwired production and perception is central to the dominant evolutionary framework, that there are six, discrete facial expressions universal across human cultures.^13^^,^^14^ However, there is a growing body of evidence that human facial expression systems are flexible and responsive to local conditions.^15^^,^^16^ For example, people are more accurate at identifying expressions from their own cultural group^17^^,^^18^ and cultural background affects visual face processing strategies.^15^ Experimental evidence suggests that prior exposure to different facial expressions can affect both peoples’ categorization of expressions and the brain regions involved,^16^^,^^19^^,^^20^ providing evidence for the malleability of facial expression processing and the potential for environmental effects. As accurate perception of facial expressions is needed to navigate complex social environments,^21^ and poor understanding can impact social relationships,^22^ health, and survival,^23^^,^^24^ a more flexible system that responds to local conditions could be adaptive.

Facial expression processing systems may be especially sensitive to environmental input during development, with some evidence suggesting that facial expression recognition typically improves with age during childhood.^25^^,^^26^ However, adverse experiences during critical periods of early social development can result in changes to facial expression processing and associated neurological processes in childhood and adulthood,^27^ specifically causing heightened sensitivity to negative expressions or general impairment in facial expression discrimination.^28^^,^^29^^,^^30^ Furthermore, children of depressed mothers (who exhibit behavioral differences from non-depressed mothers, including a decreased responsiveness to their children^31^^,^^32^) have reduced accuracy at labeling facial expressions.^33^ On the other hand, maternal sensitivity to offspring cues is positively correlated with neural responses to happy faces in the infant,^34^^,^^35^ indicating that a positive early social environment can enhance an infant’s ability to process facial expressions. Therefore, the early social environment plays a crucial role in shaping the mechanisms underlying facial expression processing in humans, with both adverse and positive experiences leaving lasting impacts.

Whether humans have evolved flexibility in facial expression systems as a recent adaptation is largely unknown. Early findings indicated that facial expression perception in non-human animals is innate, with monkeys raised in isolation responding to pictures of threat facial expressions.^36^ However, recent findings suggest a more complex picture. As they age, rhesus macaques (*Macaca mulatta*) have an increased attention bias toward threatening faces^37^^,^^38^ and respond differentially to different facial expressions.^39^ Isolation reared macaques do not respond to facial expressions in the same way as socially reared animals^40^^,^^41^ and those raised in the absence of their mother do not show typical attention bias toward lipsmacking.^42^ Furthermore, maternal rank and maternal protectiveness positively predict vigilance for threat in infant macaques,^37^ possibly as these stimuli are more novel, and macaques with abusive mothers show slower reaction times to threat facial expressions, potentially due to avoidance of these expressions.^43^ Together this evidence suggests that in macaques, early social environment can affect facial expression processing in a variety of ways, including heightening vigilance, similar to in humans.

However, severe adversity, such as maltreatment or being raised without a mother, could lead to poor development across a variety of domains and may not be a facial expression effect per se. In humans, maltreatment as a child is associated with a various deficits such as working memory, attention, and poorer language ability^44^; all of which could feed into the capacity to process faces effectively. Similarly, whether facial expression processing expertise could also be enhanced by high quality early social interactions in non-human animals is unknown. Investigating how the typical variation of face processing abilities varies in relation to social factors is important to understand the extent to which such flexibility is human unique or shared with ancient systems.

Here we investigated how maternal behavior in rhesus macaques affects facial expression processing abilities later in life. We conducted three different facial expression looking time experiments with 83 rhesus macaques aged between 1 and 16 years old and created a composite facial expression processing score. We then analyzed these scores in relation to data on maternal infant-directed behavior that individuals experienced during the first 12 weeks of life. We predicted that macaques’ facial expression processing score would improve with age, as they gain more experience with expressions, and that better quality maternal care would positively predict facial expression processing score.

## Results

### Facial expression processing: Individual experiments

**Figure 1:**
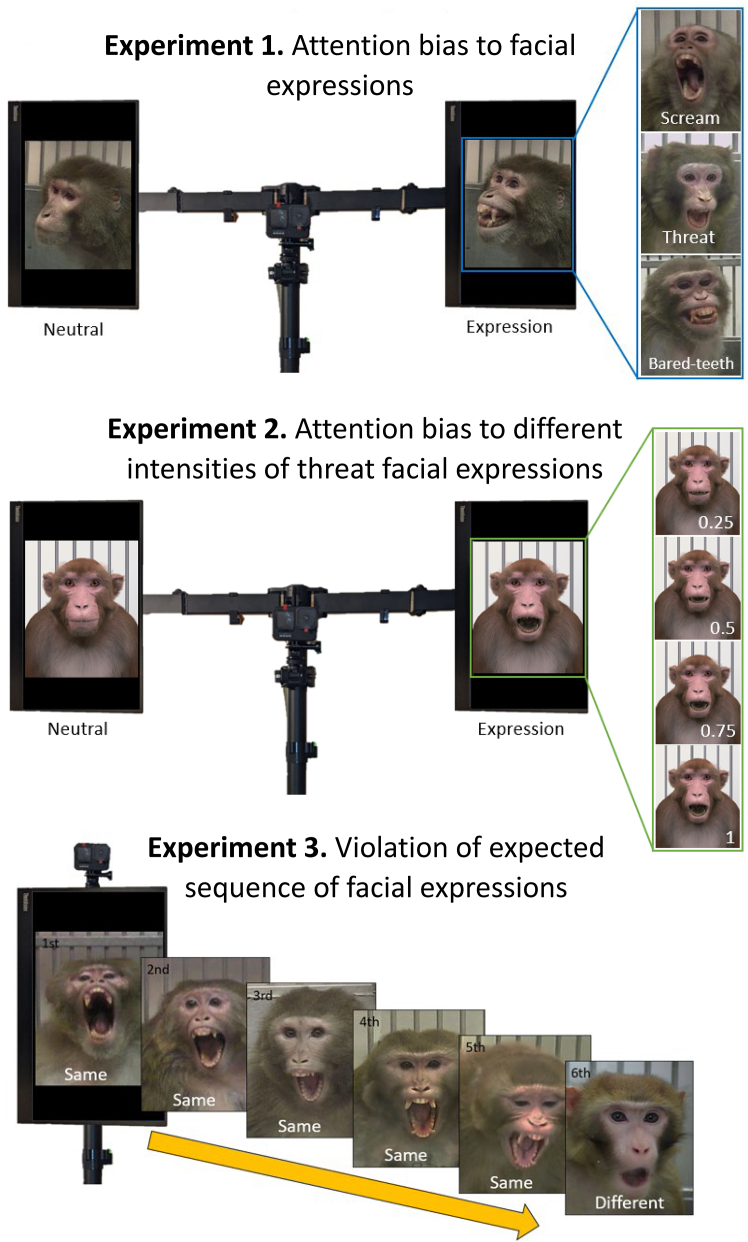
Graphical representation of the methods used in experiment 1: attention bias to facial expressions, experiment 2: attention bias to different intensities of threat facial expressions, and experiment 3: violation of expected sequence of facial expressions

We conducted three experiments, each testing different elements of facial expression processing. Experiment 1 measured macaques’ attention biases to facial expressions when compared to neutral faces, experiment 2 measured macaques’ attention biases toward threat faces of different intensities compared to neutral faces, and experiment 3 measured macaques’ abilities to distinguish facial expressions from other facial expressions and from neutral (see Figure 1).

#### Experiment 1: Attention bias to facial expressions

**Figure 2:**
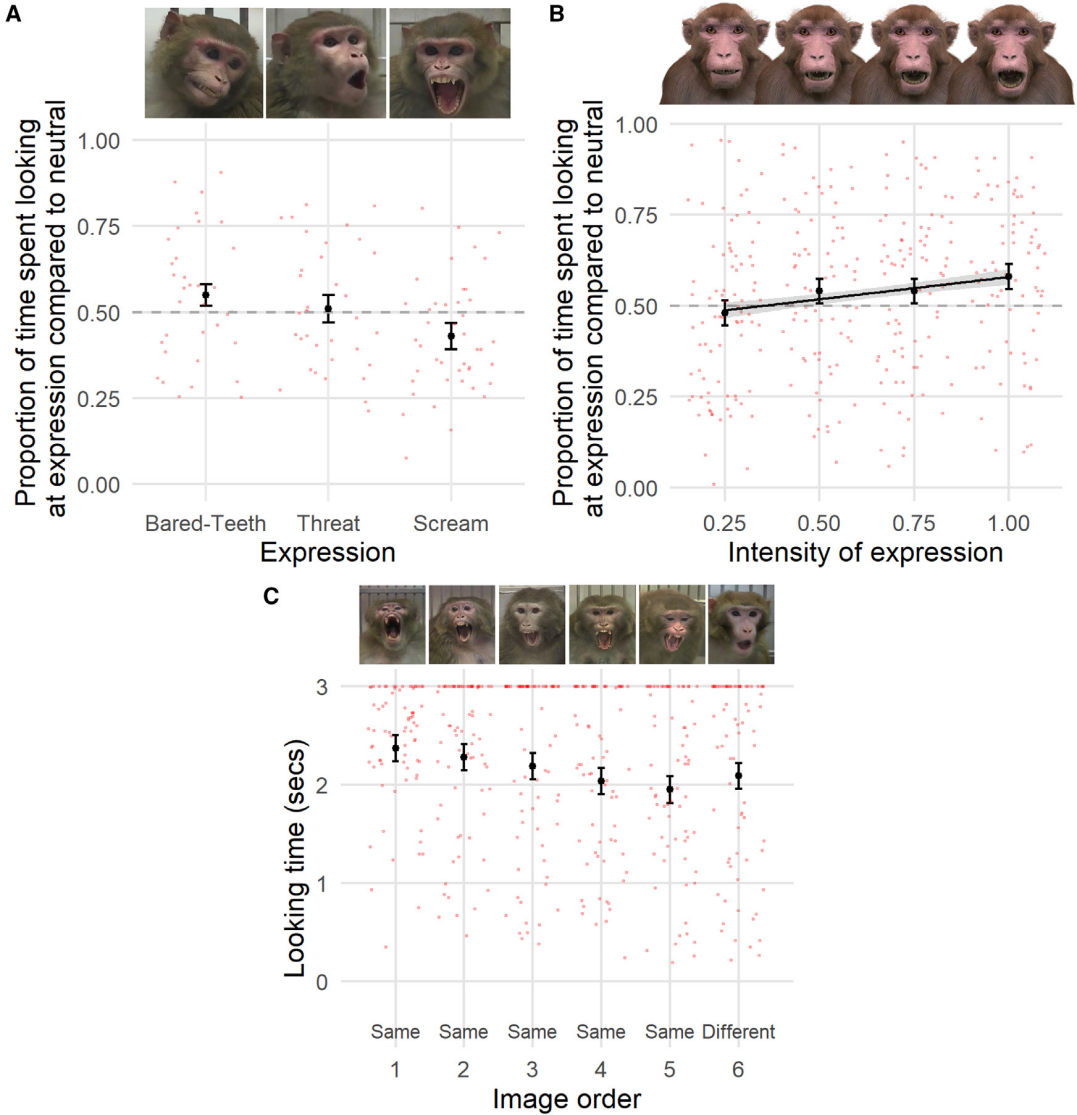
Results of facial expression processing experiments The relationship between (A) the expression type and the proportion of time spent looking at expression compared to neutral, (B) the intensity of the threat expression and the proportion of time spent looking at expression compared to neutral, and (C) the image order (the final image being different from those seen previously in the sequence) and the looking time at the image (in seconds). Black points represent predictions from the model and error bars represent the standard errors. Red dots represent the raw data and have been jittered on the x axis to improve visibility of the data density. The line in (B) represents predictions from the model where intensity is modeled as a numeric variable rather than a categorical variable, and shaded areas represent the standard errors. The dashed horizontal line in (A) and (B) highlights the point where there is no difference in looking time at the expression and the neutral face; points above the line indicate looking more at the expression than neutral, and points below the line represent looking longer at neutral than the expression.

In each trial (*n* = 112), a neutral face was displayed alongside a bared-teeth, a threat, or a scream expression. In scream trials, macaques showed a significant attention bias away from scream, with 57% of total time spent looking at the neutral face compared to 43% at the scream expression; t test: t = −2.82, df = 42, *p* = 0.007, see Figure 2A. We did not find a significant attention bias for or against the other expressions: bared-teeth (55% of looking time; t = 1.43, df = 33, *p* = 0.16) and threat (51% of total looking time, t = 0.42, df = 34, *p* = 0.68). Macaques’ responses to the bared-teeth expression and the scream expression were significantly different (55% of total looking time compared to 43%, Tukey test: bared-teeth—scream, β = 0.12, SE = 0.04, t = 2.97, *p* = 0.011).

#### Experiment 2: Attention bias to different intensities of threat facial expressions

In each trial (*n* = 313), a neutral face was displayed next to one of four different intensities of a threat expression (25%, 50%, 75%, and 100% intensity, using avatar stimuli). The intensity of the expression positively predicted the attention bias toward the expression; meaning macaques’ attention toward the threat expression increased with higher intensities of the threat expressions (linear mixed model: β = 0.12, SE = 0.04, t = 2.82, *p* = 0.005, see Figure 2B).

#### Experiment 3: Violation of expected sequence of facial expressions

In each trial (*n* = 73), a series of 5 identical facial expressions was shown, followed by a different type of facial expression, violating the expected sequence. Looking time toward the final (different) expression, was significantly higher (0.27 s higher) than would be expected based on the looking times toward previous same-type facial expressions (t test: t = 2.43, df = 72, *p* = 0.018; see Figure 2C).

### Facial expression processing: Individual differences

**Figure 3:**
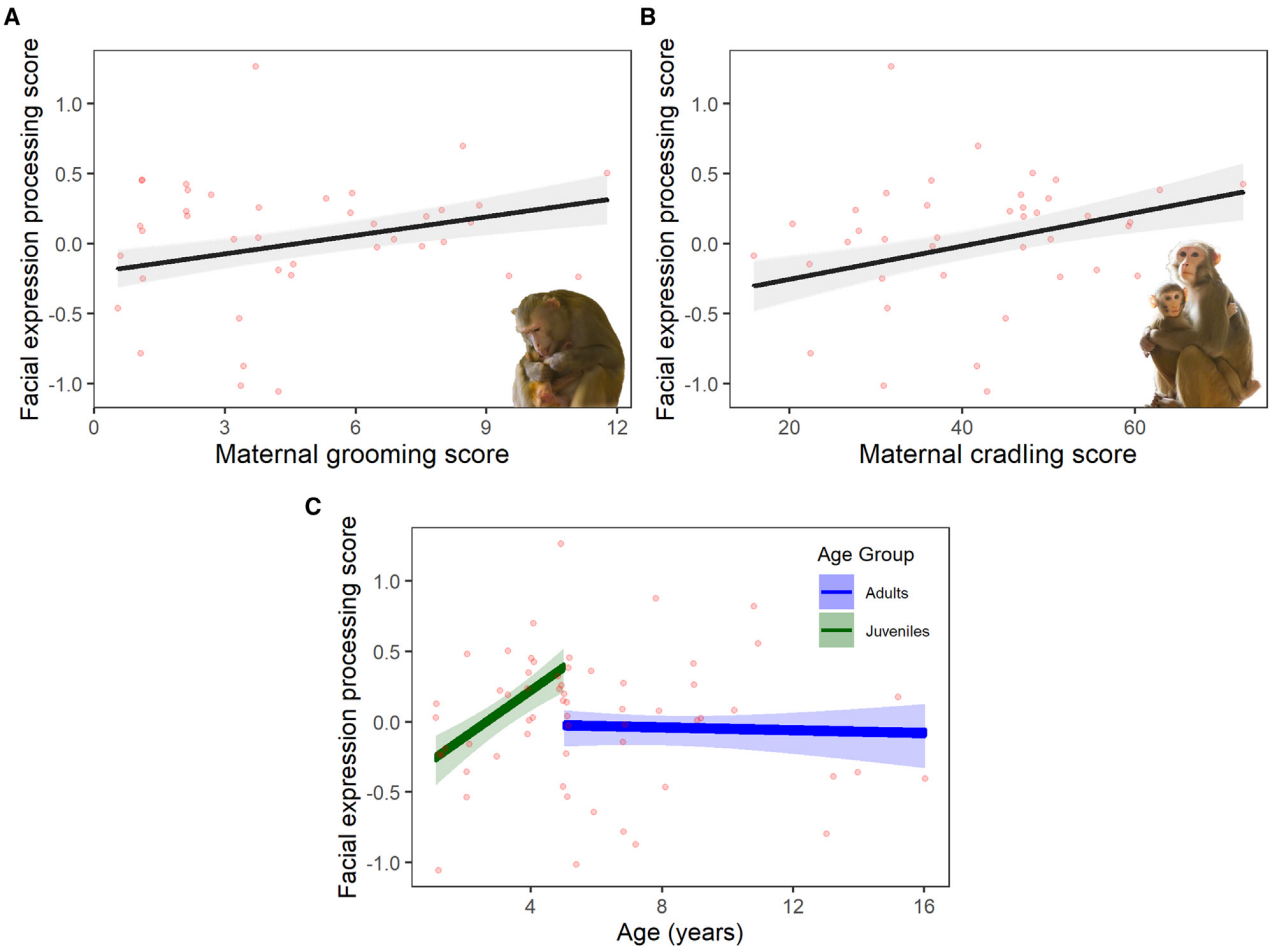
Factors imacting facial expression processing score The relationship between (A) maternal grooming score and the facial expression processing score, (B) maternal cradling score and the facial expression processing score, (C) age (continuous), age group (categorical), and the facial expression processing score from the follow up age-study. In (C) the blue line and shaded area represent adults and the green line and shaded area represent juveniles. Lines represent predictions from the model, while shaded areas represent the standard errors.

We created a composite facial expression processing score for each individual, based on their performance across the aforementioned experiments. A linear model showed that two maternal care behaviors, maternal grooming and maternal cradling, both had a positive effect on facial expression processing score (see Figures 3A and 3B; Table 1). The model also showed that age had a quadratic relationship with facial expression processing score, with age having a positive effect between the ages of 1 and ∼4, and then a negative one from ∼4 to 7 (See Table 1). We conducted a follow up analysis to clarify this effect using a larger sample, involving older individuals without maternal data (up to 16 years old), which showed no significant effect of age, polynomial age or log(age). However, when age as a categorical variable (where individuals are split into juveniles [under 5] and adults [5 and over]) was added into the model, a significant interaction emerged between continuous age and this categorical age, with age having a positive effect on facial processing score for juveniles, and no effect for adults (continuous age∗categorical age: juvenile: β = 0.17, SE = 0.07, t = 2.53, *p* = 0.015, see Figure 3C).

**Table 1:** Results of linear mixed effects model

Coefficients	Estimate	Std. error	df	t value	*p* value
(Intercept)	−0.790	0.291	28.491	−2.716	0.011∗
Grooming	0.044	0.021	29.557	2.086	0.046∗
Cradling	0.012	0.006	27.053	2.070	0.048∗
Approaching	1.426	0.792	22.528	1.802	0.085
Restraining	−0.640	0.748	31.921	−0.856	0.398
Sex (male)	−0.007	0.126	25.008	−0.052	0.959
poly(age, 2)1	0.297	0.459	31.917	0.648	0.522
poly(age, 2)2	−1.400	0.403	31.903	−3.475	0.001∗

Significant *p* values are marked with asterisks.

## Discussion

The population level analysis of our three experiments showed that rhesus macaques can distinguish between certain facial expressions and respond differentially to varying intensities of the same expression. Individuals, however, differed in their performance on these tasks. We found that macaques who had experienced higher quality maternal care (more grooming and cradling) during infancy, had better facial expression processing skills. This provides evidence that maternal care facilitates the development of facial expression processing skills in non-human animals (as in humans^33^^,^^34^^,^^35^). We also found that individuals’ facial expression processing proficiency increased with age until around 4 years old, suggesting that these skills may reflect experience and environmental input, similar to humans.^26^^,^^45^^,^^46^ Our results provide evidence that facial expression perception is not innate in macaques and is instead influenced by the quality of social input.

In our analysis of maternal behavior on facial expression processing score, the relationship between age and facial expression processing abilities showed a quadratic pattern, with skills improving up to around 4 years of age and then declining until 7 years of age (the maximum age in this dataset). However, further analysis with a larger sample including older individuals without maternal data (up to 16 years old) revealed a more nuanced picture, that juveniles (under 5 years) showed improvement with age while adults (5 years and older) showed no significant age-related changes. The initial quadratic relationship may have been an artifact of the limited sample, mistaking the transition from rapid juvenile improvement to adult stability as a decline. The pattern we observe in the follow up age-analysis suggests that facial expression processing abilities may develop rapidly during early life and then stabilize in adulthood, as is the case with several cognitive abilities in humans.^47^ Studies in humans find that facial expression discrimination abilities increase through childhood,^25^ with performance in adulthood staying constant for some expressions but decreasing for others.^48^^,^^49^ This increase and then plateau in facial expression processing score with age could be a product of changes in interest in facial stimuli; for example, humans experience a decrease in attention to faces in early life.^50^ However, interest in faces and facial expressions is also an important element of facial expression processing, so the two explanations are related.

A possible explanation for why maternal grooming and cradling could have a positive effect on facial expression processing skills is that these behaviors involve close, often face-to-face, contact between mother and infant. In a study on non-human primates, mother-infant mutual gazes were longer during caring and grooming (as well as feeding, compared to other behaviors including locomotion, resting, and play;^51^). Newborn rhesus macaques, like humans, are attracted to faces and eyes,^52^ especially those with direct gaze,^53^ and mother-infant face-to-face interactions are shown to be beneficial to macaque social development.^54^ During face-to-face interactions, macaque mothers often lipsmack at their offspring,^55^ which provide offspring with opportunities to observe and learn facial expressions, and to develop increased sensitivity to facial movement more generally (which would also explain increased detection of perhaps lesser experienced expressions such as threat) In addition, macaque mothers gaze more at the faces of sons than daughters,^56^ leading to sons (but not daughters) looking significantly more at eyes when viewing facial expressions,^57^ which is considered important for facial expression processing.^58^ This suggests that maternal face-to-face contact plays an important role in the development of infant facial expression processing skills. We, however, find no sex differences in our study, so the effects of maternal gaze leading offspring to gaze more at eyes when viewing facial expressions may not be driving facial expression processing skills.

Face-to-face contact, as well as providing opportunities for infants to observe their mother’s facial expressions, could also facilitate them socializing with other group members, which could allow them to encounter more facial expressions and in more diverse social situations. Infant macaques who have more face-to-face interactions with their mothers also have more social interactions at 2 and 5 months old.^54^ This effect was unlikely to be due to physical contact alone, as nursery-reared monkeys who received controlled face-to-face interactions showed increased social interest (spending more time looking at social stimuli than non-social stimuli, and more time interacting with peers), while those who received just physical handling did not. Furthermore, mother-reared (unlike nursery-reared) infants show an attention bias toward facial expressions (lipsmacking), with greater bias toward these expressions being related to higher levels of social engagement.^42^ This could suggest that maternal presence facilitates infants’ attention bias toward facial expressions through facilitating social engagement with others. A possible mechanism by which this maternal care could facilitate social interactions, either through face-to-face contact, or through the physical touch itself, is by increasing oxytocin levels in the offspring^59^^,^^60^^,^^61^ or by reducing infant cortisol levels.^62^ Specifically, administered oxytocin promotes positive social behavior and increases attention and response to faces (with facial expressions), suggesting a positive impact of oxytocin on facial processing.^63^

Beyond facilitating face-to-face interaction, there may be other explanations as to why specifically grooming and cradling behaviors from the mother are driving this effect. Individuals who are groomed frequently may be healthier, due to the hygienic significance of grooming.^64^ Individuals in better physical health may be afforded other benefits, such as increased social interaction and engagement with others. Individuals spending an increased amount of time cradling their mother are likely to be a spectator of more social interaction on the whole; having an increased opportunity to closely observe their mothers’ interactions as a third party (which are likely to include both affiliative interactions and conflict).

If facial expression processing skills are flexible, it is possible that the variation in facial expression processing skills represent a facultatively adaptive response to environmental conditions. It is possible that better facial expression processing skills are advantageous in some social environments and not in others, so different early life environments could provide cues for different strategies. For example, a paucity of maternal care could indicate an unstable or resource-poor environment if mothers must trade off time between maternal care and survival,^65^ which may cue offspring to give developmental priority to skills that are more immediately relevant to survival in such an environment.^66^ In which case it may be more adaptive to develop heightened responses toward resources, or immediate threats, than toward a variety of facial expressions. Past research has shown that rhesus macaque males with more diverse facial behavior are better connected to their social group; however, those with less diverse facial behavior win a greater number of competitive interactions.^67^ This suggests that different levels of facial expressivity could better equip individuals for different niches and the same could be true for facial expression perception. However, if levels of maternal care can be a reliable cue to the environment, and indeed whether reduced facial expression processing skills are ever adaptive, remain to be tested. It is also possible that there are more nuanced facultative responses to early social adversity than we mention here, such as increased response to threat expressions.^68^ This could be the case even if in such environments overall facial expression discrimination skills are reduced. Studies exploring the adaptive value of facial expression have demonstrated that expressivity is explained by both social and parental styles^69^^,^^70^ in primates. At a species level, those individuals with less despotic social systems^71^ and increased alloparenting^72^ have increased complexity in their facial behavior (i.e., more muscle dexterity and higher rates of facial behavior). This is suggested to be in response to the increased complexity of the social interactions they engage in and the more differentiated social relationships that they need to maintain. Similarly, the variation we see in facial processing may be individual responses to social complexity; those individuals with more social (and more tolerant) mothers may require more sophisticated processing tools to successfully navigate their social environment compared with those with less social mothers.

We find that facial expression processing skills improve with age until adulthood and are positively associated with maternal care. These findings provide evidence for the flexibility of facial expression processing, suggesting that rather than being entirely hardwired, its development requires environmental input. As a result, facial expression processing has the potential to adapt to different environmental conditions. Our results help to demonstrate that such flexibility in facial expression processing is not unique to humans, but likely emerged earlier in our evolutionary history.

### Limitations of the study

There are some limitations impacting this study that should be taken into account when evaluating the findings. First, as is common in developmental research, the age-related findings are based on cross-sectional data from different individuals. Therefore, while we can see age-related differences in facial expression processing in a large sample of individuals, we have not conducted a longitudinal analysis of the same animals over time. Second, our findings are based on animals in a captive laboratory setting. This setting facilitates the use of controlled, experimental methods that are necessary to assess cognitive skills in facial expression processing, but whether our findings are generalizable to animals in a wild environment is yet to be tested.

## Resource availability

### Lead contact

Further information and requests for resources should be directed to and will be fulfilled by the lead contact, Olivia O’Callaghan (olivia.o’callaghan2021@my.ntu.ac.uk).

### Materials availability

This study did not generate new unique reagents.

### Data and code availability

• All original data have been deposited at Open Science Framework at https://osf.io/7hvjd and is publicly available as of the date of publication.
• All original code has been deposited at Open Science Framework at https://osf.io/7hvjd and is publicly available as of the date of publication.
• Any additional information required to reanalyze the data reported in this paper is available from the lead contact upon request.

## Acknowledgments

We thank Kerensa Rees and Natalia Ptaszynska for help coding the videos and Katalin Gothard for permitting us to use her video recordings to create our stimuli. We would like to thank all staff of the Center for Macaques, and in particularly the husbandry and technical staff. This project has received funding from the 10.13039/501100000781European Research Council (ERC) under the 10.13039/501100007601European Union’s Horizon 2020 research and innovation program (grant agreement no. 864694 to B.M.W.). This work was also supported by the 10.13039/501100000265Medical Research Council (Award MC_UP_1504/1).

## Author contributions

O.O., conceptualization, raw data collection, data analysis, writing (original draft); J.W., conceptualization, raw data collection, writing (review and editing); A.P., conceptualization, writing (review and editing); C.L.W., conceptualization, project administration, writing (review and editing); B.M.W., conceptualization, supervision, funding acquisition, project administration, writing (review and editing).

## Declaration of interests

The authors declare no competing interests.

## STAR★Methods

### Key resources table

**Table:** 

REAGENT or RESOURCE	SOURCE	IDENTIFIER
**Deposited data**		

Original data deposited for this study	Open Science Framework	https://osf.io/7hvjd

**Experimental models: Organisms/strains**		

Rhesus macaques (*Macaca mulatta*)	Center for Macaques, Medical Research Council. *N* = 83 Animals (*n* = 56 females, *n* = 27 males; *n* = 45 juveniles, *n* = 38 adults). Animals were allocated to experimental conditions randomly. All animals were used in research activities approved by the AWERB of Center macaques: CFM2022E001). Each animal had access to two adjacent rooms: one indoor room (3.5 × 8 × 3m), with a large outdoor-facing window, enriched with climbing structures, feeding puzzle boxes, and other enrichment devices, and a second indoor caged area (1.5 × 6 × 3m). Their diet consisted of commercial monkey pellets, fruits, and vegetables, supplemented with a scatter feed of dried forage mix. Water was freely available.	

**Software and algorithms**		

BORIS: Behavioral Observation Research Interactive Software	Friard and Gamba^73^	https://www.boris.unito.it/
ICC R package	Wolak et al.^74^	https://cran.r-project.org/web/packages/ICC/index.html
R: A language and environment for statistical computing	R Core Team ^75^	http://www.r-project.org/
lme4 R package	Bates et al.^76^	https://cran.r-project.org/web/packages/lme4/index.html
emmeans R package	Lenth^77^	https://cran.r-project.org/web/packages/emmeans/index.html
Animal Observer	Caillaud and Observer^78^	https://fosseyfund.github.io/AOToolBox/

**Other**		

Avatar stimuli	Murphy and Leopold^79^	https://figshare.com/authors/Aidan_Murphy/6796655

### Experimental model and study participant details

Subjects consisted of 83 rhesus macaques (*Macaca mulatta;* 56 female) from 15 different social groups, with a mean of 9.15 non-infants per group (range: 6–12). Groups were either breeding groups, comprising one adult male, 3-8 adult females, and their offspring; or single-sex groups. Experimental subjects were at least 1 year old. All animals were housed at the Medical Research Council’s Center for Macaques (CFM), Salisbury, UK, in uniform enclosures. Each animal had access to two adjacent rooms: one indoor room (3.5 × 8 × 3m), with a large outdoor-facing window, enriched with climbing structures, feeding puzzle boxes, and other enrichment devices, and a second indoor caged area (1.5 × 6 × 3m). Their diet consisted of commercial monkey pellets, fruits, and vegetables, supplemented with a scatter feed of dried forage mix. Water was freely available. The research received ethical approval from the CFM AWERB (Animal Welfare and Ethical Review Body) on the 3rd February 2022 with the reference number CFM2022E001.

### Method details

#### Data collection

##### Cognitive experiments

A battery of three different experiments were carried out between April 2022 and September 2022 by two experimenters (OO and JW). Each experiment was designed to test facial expression discrimination in a different way, experiment 1 tests discrimination of expressions from neutral, by utilizing the macaques’ attention biases. Experiment 2 tests discrimination of expressions of different intensities from neutral, by utilizing the macaques’ attention biases. Experiment 3 tests discrimination of expressions from other expressions and from neutral; however it is not reliant on their being attentional differences toward different expressions, but solely on the macaques’ recognition that the two expressions are different. Each experimental session was conducted outside the macaques caged room. Macaques could move freely between this room and a second room throughout all experiments and as such there were often multiple macaques present during each trial. A trial would start when a macaque had positioned themselves in front of the equipment at a distance of between 0.5 and 1 m. Participation was voluntary, though food incentives (raisins and peanuts) were offered to encourage macaques to position themselves in front of the equipment. Participants were presented with the three different experiments in no particular order. Each group typically received no more than 1 experimental session per week and macaques received no more than 2 trials per session. Research shows that rhesus macaques’ attention to a looking task decreases with daily participation but not with weekly participation^80^ and therefore participation was limited in line with these data.

###### Equipment and stimuli

For experiments 1 and 2, the equipment consisted of two digital screens (14″), positioned 65cm apart (center to center), with a camera between them. For experiment 3, one digital screen (14″) with a camera above it (to record the monkeys gaze direction) was used. All screens were displayed 50cm above the macaques’ floor level. All stimuli were color images (21cm in height and 17cm wide). Stimuli for experiments 1 and 3 depicted photographs of real unfamiliar macaques, and stimuli for experiment 2 depicted digital avatars.^79^ Stimuli used within a trial were adjusted to be consistent with each other in brightness and contrast. We were not able to control the intensity of the expressions of the stimuli used in Experiment 1 or 3 as we were in Experiment 2 as they are from real macaques rather than avatars, however we selected our stimuli on the basis of them having high intensity expressions. We estimate them to all be between 75% and 100% intensity. Lip smacking faces were not used in any of our experiments as, although all facial expressions are dynamic, lip smacks are considered to be especially hard to represent in still images, due to their rhythmic nature and accompanying sound. For example, past studies have found attentional bias toward lip smacking in rhesus macaques using video clips^38^ but not using still images.^42^

###### Experiment 1. Attention bias to facial expressions

Experiment 1 was an attention bias task, where two faces were presented simultaneously, one on each screen. In each trial, a neutral face was shown on one side (either the left or right), and an expression (either bared-teeth, threat or scream) was shown on the other, with both images being of the same individual (see Figure 1). We interpret a looking bias, either toward or away from an expression, as evidence of perception that it differs from the neutral face. Images were shown for 5 s, followed by 0.5 s of a black screen, before the images were shown on the opposite side (to reduce the effect of possible side biases). An audible cue (a beep) occurred when the images appeared and again when they swapped sides as a reference to the researcher during video coding. There were 3 possible stimuli pairs for each expression (i.e., 9 trials in total) that varied whether the expression started on the left or the right. For individuals’ first trial, they were randomly assigned one of the 9 trials, for their second trial, they were randomly assigned one of the trials of the remaining two expression types, and for their third, they were randomly assigned one of the trials of the one remaining expression type. This process would then be repeated, excluding the specific trials they had already received.

###### Experiment 2. Attention bias to different intensities of threat facial expressions

Experiment 2 compares different intensities of a single expression type to a neutral face using an avatar.^79^ Threat expressions were used as the expression type based on previous findings that threats provoke looking time differences in attention bias experiments (e.g., 61). Stimuli depicting four different intensities were generated: 100% intensity, 75% intensity, 50% intensive, and 25% intensity (see Figure 1). We interpret a difference in looking time between more subtle versions of the expression and neutral as a sign of facial expression processing expertise. The design is similar to that of experiment 1, however for each expression intensity there is only one pair of stimuli (which was swapped to vary which side the expression started on). Although using avatars means stimuli are potentially less naturalistic, it allows for more control over expression intensity.

###### Experiment 3. Violation of expected sequence of facial expressions

Experiment 3 was a violation of expectation task, where five different photographs of the same facial expression were presented in succession, followed by a photograph of a different type of facial expression, thereby violating the expected sequence (see Figure 1). Threat, scream and neutral faces were used as the stimuli for the initial sequence of five same-expression-type faces, while threat, scream and bared-teeth were used as the final, sequence violating images. Bared-teeth was never used for the initial sequence due to a lack of stimuli of this type, while neutral was never used as the final, sequence-violating image, as we wanted this to always be an expression. We interpret looking longer at the final image as evidence that a macaque recognizes that this sequence-violating expression is different from those seen earlier in the sequence. Photos were shown for 3s each, with a half a second gap, in which the screen was black, in between, and a sound cue accompanying the presentation of each image.

###### Video coding

The videos captured during the experiments were coded if they met inclusion criteria: for experiments 1 and 2, the macaque had to have looked at both the expression and neutral image and for experiment 3, the macaque had to have looked at at least four of the five initial images and the final image. There are the fewest number of trials for experiment 3 as macaques were less likely to maintain concentration throughout this trial as it required attention to successive images. 177 videos (across the three experiments) did not meet these criteria and were thus not coded. Videos were coded frame by frame in BORIS^73^ for the macaques’ looking direction: left or right screen for experiments 1 and 2, and at the screen for experiment 3. Coders were blind to the trial type. To assess intercoder reliability, we employed the intraclass correlation coefficient (ICC) using the ICC function from the *psych* package in R.^74^ Nine videos were randomly selected and independently coded by the two coders. The analysis revealed strong agreement, with an average ICC of 0.91 (CI: 0.68–0.97, *p* < 0.001) across codes.

#### Maternal behavior data

Maternal behavior data were collected between January 2015 and November 2022 as part of routine husbandry at the CFM. For each infant, nine 10-min focals were carried out during their first 14 weeks of life, which were distributed equally across three time periods, when the infant was: 0–2, 6–8 and 12–14 weeks old. Four measures are used in the analysis: two derived from scan samples (which were carried out every 30 s and recorded the type of contact between mother and infant), grooming and cradling, and two derived from focal observations, mother approaching infant (moving to within 1m of infant) and mother restraining infant (when infant tries to break contact with mother but she restrains them). (For more detail on maternal behavior data, see Methods S1: Maternal behavior data (for validation)).

### Quantification and statistical analysis

All statistical analyses and visualizations were carried out using R version 4.1.2^75^ and linear mixed models (LMMs) were run using lme4.^76^

#### Facial expression processing: Individual experiments

To analyze experiment 1 (attention bias to facial expressions), we ran an LMM with attention bias as our response variable (the time spent looking at the expression as a proportion of the total time spent looking at both images). The fixed effect was expression type (threat, scream and bared-teeth) and random effects were group and individual ID, with individual ID nested within group. To determine which expressions were responded to differently, we computed pairwise comparisons of the estimated marginal means for the attention biases for the different expressions using the emmeans function from the emmeans package.^77^ To evaluate whether the proportion of time spent looking toward each expression differed significantly from 0.5 (i.e., if there was an attention bias), we performed a one-sample t-test for each expression.

To analyze experiment 2 (attention bias to different intensities of threat facial expression), we ran an LMM with attention bias as our response variable. The fixed effect was expression intensity (as a continuous numeric variable) and random effects were group and individual ID, with individual ID nested within group.

To analyze experiment 3 (violation of expected sequence of facial expressions), we fitted a linear model using the looking times at the first five images (those that are shown prior to the different sequence-violating expression) and used it to predict the expected looking time for the 6th image. We then compared the predicted looking times with the actual observed looking times for the 6th image using a paired t-test.

#### Facial expression processing: Individual differences

In order to study the individual differences in macaques’ facial expression processing, we made a composite facial expression processing score for each individual, based on their performance in our three cognitive tasks. This score primary measures discrimination ability, with higher scores indicating a macaque is more able to discriminate between expressions/between expressions and neutral. From experiment 1: attention bias (the time spent looking at the expression as a proportion of the total time spent looking at both images), was transformed to absolute values, so that attention bias toward or away from expression are treated equally. From experiment 2: attention bias to different intensities of threat, we use the attention bias to contribute to the composite facial expression processing score. From experiment 3: violation of expected sequence of facial expressions, we use the binary response of whether or not the actual observed looking time at the final sequence-violating image is higher than the looking time predicted by the looking time at the previous 5 images. To make the composite score, for each different trial type (e.g., Experiment 1, trial type: bared-teeth), the scores mentioned above were standardized to z-scores and then they were averaged across all trial types for each individual to produce their composite facial expression processing score. Individuals were used in our analysis if they had completed at least 3 trials in total, which could be from any of the three experiments. Individuals’ scores therefore vary in how many trials they have contributing to them and the distribution of these trials among the different experiment types. It is worth noting that individuals vary in how many trials they completed, and how these trials were distributed between the different experiment types, so each individual’s facial expression processing score is composed of a different combination of trials and thus is not perfectly comparable.

Using this score as the response variable, we ran an LMM, including only macaques who we had maternal behavior data for (40 individuals, 24 female, from 12 different groups). Fixed effects were sex and a polynomial term for age, to account for potential quadratic relationships between age and facial expression processing score. Fixed effects also included maternal behaviors: maternal grooming score, i.e., the proportion of scans during which the individual was being groomed by their mother when they were an infant; maternal cradling score, i.e., the proportion of scans during which the individual’s mother had her arms round them when they were an infant; maternal approaching score, i.e., the number of times their mother approached them when they were an infant; and maternal restraining score, i.e., the number of times their mother restrained them when they tried to leave her contact when they were an infant. Random effects were group and mother ID.

To further investigate the effects of age on facial expression processing score, we did a follow up analysis with the inclusion of additional individuals who had done at least 3 trials but did not have maternal behavior data. This analysis included 60 individuals, 37 females. Four different LMMs were ran, each had facial processing score as the response variable, sex as a fixed effect and group as a random effect. Each model had a different specification of age as the fixed effect(s): age, log(age), age as a polynomial term, to account for a potential quadratic relationship, and age as both a continuous variable and a categorical variable (where individuals are split into juveniles (under 5) and adults (5 and over)).

## Supplemental information

Document S1. Method S1
