# Supplementary material for: Maternal care predicts facial expression processing in macaques
Source: iScience. 2025 Mar 25;28(4):112179. doi: 10.1016/j.isci.2025.112179 (PMC12131261; doi:10.1016/j.isci.2025.112179)
Supplement: Document S1. Method S1 [file mmc1.pdf]

iScience, Volume *28*

Supplemental information

Maternal care predicts facial expression processing in macaques

Olivia O'Callaghan, Jamie Whitehouse, Annika Paukner, Claire L. Witham, and Bridget M. Waller

Methods S1: Maternal behaviour data (for validation), related to STAR methods

Maternal behavioural data on 8 infants (selected at random) were collected between April 2022 and September 2022 in order to verify whether the cross-sectional maternal behaviour data collection methods used at the CFM provide a representative overview of maternal behaviour. Behaviours recorded include, those recorded via focal sampling: restraining the infant, making and breaking contact with the infant and approaching and leaving the proximity (1m radius) of the infant, and those recorded through scan sampling (which was every 30 seconds) and included the type of contact (grooming, cradling, no contact, and other) and distance between the infant and mother. For our validation study, the same behaviours were recorded as outlined above, however, each infant was observed for a longer period of time, an average of 188 mins (range: 117-225) compared to 90 minutes across the first 14 weeks, and data was collected using the application Animal Observer1. We compared the data we collected on each of the 8 infants with the data the CFM had collected on those same infants during the same time period. For each behaviour we tested the correlation between the scores obtained from the routine data collection at the CFM and from the more intensive data collection. We chose to use the following behaviours due to their high correlations: For mother approaches infant, we found a correlation of 0.75, mother restrains infant: 0.90, mother cradles infant: 0.74, mother grooms infant: 0.65. We chose not to use the following behaviours due to their low correlations: mother makes contact: -0.01, mother leaves infant: 0.15, mother refuses contact with infant: 0, mother breaks contact with infant: 0.03.

1. Damien Caillaud, Animal Observer. A free iPad application for behavioral observations, activity budgets and animal health monitoring. (2012).
